# Extended difficulties following the use of psychedelic drugs: A mixed methods study

**DOI:** 10.1371/journal.pone.0293349

**Published:** 2023-10-24

**Authors:** Jules Evans, Oliver C. Robinson, Eirini Ketzitzidou Argyri, Shayam Suseelan, Ashleigh Murphy-Beiner, Rosalind McAlpine, David Luke, Katrina Michelle, Ed Prideaux

**Affiliations:** 1 Centre for the History of Emotions, Queen Mary University, London, United Kingdom; 2 School of Human Sciences, University of Greenwich, London, United Kingdom; 3 Psychology Department, University of Exeter, Exeter, United Kingdom; 4 Department of Psychology, Royal Holloway, London, United Kingdom; 5 Division of Psychology & Language Sciences, University of College London, London, United Kingdom; 6 Department of Applied Psychology, New York University, New York, New York, United States of America; 7 Researcher, Perception Restoration Foundation, San Juan, Puerto Rico, United States of America; University of Brescia: Universita degli Studi di Brescia, ITALY

## Abstract

Long-term adverse experiences following psychedelic use can persist for weeks, months, or even years, and are relatively unexplored in psychedelic research. Our convergent mixed-method study gained quantitative and qualitative data from 608 participants who reported extended difficulties following psychedelic experiences. Data was gathered on the context of use, the nature and duration of the challenges they experienced (including a written description of these), plus a range of possible risk factors and perceived causes. The most common forms of extended difficulty were feelings of anxiety and fear, existential struggle, social disconnection, depersonalization and derealization. For approximately one-third of the participants, problems persisted for over a year, and for a sixth, they endured for more than three years. It was found that a shorter duration of difficulties was predicted by knowledge of dose, drug type and lower levels of difficulty reported during the psychoactive experience, while a narrower range of difficulties was predicted by taking the drug in a guided setting. Implications for psychedelic harm reduction are discussed.

## Introduction

Psychedelic drugs have been consumed for thousands of years by humans in many cultures for religious worship, healing, magic, recreation or personal development [1]. Indigenous cultures in Africa and the Americas developed rituals and terminology for these substances that survive today [2]. Western culture rediscovered these drugs in the late 19th to mid-20th century, calling them ‘hallucinogens’, ‘entheogens’ or, most popularly, ‘psychedelics’ [3]. The first major wave of psychedelic research in the 1950s-60s ended disappointingly, with the FDA declaring there was no medical benefit and serious public health risks to these substances [4]. The latest wave of research and investment—the so-called psychedelic renaissance—has seen a revival of optimism that psychedelic drugs hold great therapeutic and spiritual potential [5].

Interest in psychedelic drugs in western countries has grown rapidly in the last few years, both in academic research, media attention and public usage. Data from a 2023 report from the USA shows that 8% of US adults aged 19–30 reported past-year usage of psychedelics in 2022, compared to 3% in 2012. US adults aged 35–50 reported even higher growth in usage, from less than 1% in 2012 to 4% in 2022 [6]. Most of that usage happens in non-medical underground settings, for a range of motives–including healing, self-exploration, spiritual growth and fun.

Many US states and cities have decriminalized some psychedelic drugs since Denver first decriminalized magic mushrooms in 2019, and California is poised to approve similar legislation [7]. Meanwhile, clinical trials for psychedelic therapy are close to gaining FDA approval in the US, and therapy involving MDMA or psilocybin is already available in Australia as of 2023 [8].

While there is mounting evidence of the therapeutic benefits of psychedelic drugs when taken in a clinical setting (and also outside of a clinical setting) there is less research on the harms that people can experience during and after psychedelic experiences, or what helps them to cope with those harms.

Adverse effects of psychedelics (unwanted, negative, distressing experiences), that occur during or after the acute psychedelic experience, are generally poorly defined in the field and may be underreported [9, 10]. De Laportalière et al. conducted a systematic review of adverse events reported in clinical trials of esketamine (a form of ketamine) for depression [11]. They found that 41.5% of serious adverse events and 39% of non-serious adverse events went unreported in publications of study findings. McNamee et al. raised concerns about the lack of research on psychedelic harms and call for further research, including phenomenological studies on adverse outcomes, to address the issues around informed consent [12].

### Extended adverse effects and experiences

There is a growing body of research on challenging experiences during the acute psychedelic experience [13], and a validated measures of how intense the acute challenging experience is [14]. The phenomenology of the acute experience includes fear, grief, feelings of insanity, paranoia, isolation, death, physical distress, confusing and troubling visions [13, 14]. As a matter of terminology, in this article, we will mainly use the more colloquial terms ‘trip’ or ‘psychedelic trip’ rather than acute psychedelic experience, for the sake of brevity.

In contrast to the growing literature on acute difficult experiences, research on adverse experiences that endure *after* the pharmacological effects of psychedelics is notably limited. In Carbonaro’s study of psilocybin mushrooms’ adverse effects, 24% of the participants reported experiencing one or more symptoms (anxiety, paranoia, depression, fear), that lasted a week or longer after the psychedelic session, and which they attributed to the psilocybin experience [15]. 10% reported psychological symptom(s) lasting more than a year after the challenging psychedelic experience, with 7.6% seeking professional treatment for the symptom(s).

In Simonsson and colleagues’ analysis of challenging, difficult, or distressing experiences using classic psychedelics, 6.7% of participants reported thoughts or attempts of hurting themselves or others, 4.6% disclosed thoughts of hurting oneself; 2.6% reported thoughts of hurting others; 1.5% admitted to attempts to self-harm; and 2.6% sought medical, psychiatric, or psychological assistance in the days/weeks following their most distressing psychedelic experience [16]. In findings from the Global Ayahuasca survey, 12% reported lasting adverse effects for which they sought professional support [17]. In the 2020 Global Drug Survey of LSD and psilocybin, 22.5% of the total sample reported at least one negative outcome, with the most common being “mental confusion, memory problems, or racing thoughts”. 6% of the total sample reported difficulties lasting longer than one month. 0.9% of the total sample sought medical help following self-treatment with LSD or psilocybin [18].

Transient visual distortions experienced after taking a psychedelic substance have been reported by 40–60% of users [19, 20]. This is not a phenomenon specific to psychedelic users as it can also be caused by alcohol or benzodiazepines or even occur in healthy populations [21]. In cases where these symptoms are prolonged and distressing, they form a syndrome known as Hallucinogen Persisting Perception Disorder (HPPD), recognized by the DSM-V. In one study, 4.2% of participants said they experienced continued hallucinations after a psychedelic trip that were troubling for them [19]. However, HPPD prevalence rate remains unclear [22–24]. Halpern and colleagues suggest that pre-drug use complaints of tinnitus, eye floaters and concentration problems, or a personal/family history of anxiety, may increase vulnerability to developing HPPD [21]. It is possible that HPPD represents a form of traumatic anxiety disorder akin to PTSD [21, 25] or a form of health anxiety [26] triggered by the residual symptoms of the psychedelic experience.

Bremler et al. interviewed individuals experiencing long-term negative effects from psychedelic use and found that 80% reported experiencing a sense of similarity or connection to an unpleasant psychedelic experience [27]. Some referred to this re-experiencing as ‘flashbacks’ or ‘emotional flashbacks’ and some reported re-experiencing physical symptoms such as nausea they had experienced during the original psychedelic trip. Almost half of the interviewed participants (46.7%) described feelings of disconnection and isolation [27]. The same number of participants described derealization experiences, including feeling out-of-body and an altered sense of reality around them, sometimes described as ‘losing connection with reality’.

The association between prolonged psychosis risk and psychedelic use has been a topic of long-standing debate, yet the evidence establishing links remains inconclusive [28–30]. According to Bogenschutz and Ross, older reports of LSD-induced psychosis prompted modern psychedelic researchers to exclude participants with a personal or family history of psychotic spectrum illness, leading to low prevalence in more recent psilocybin or LSD clinical trials [31]. Carbonaro et al. did identify three cases who reported enduring and impairing psychotic symptoms following a challenging psilocybin experience [15]. Dos Santos et al. undertook a review of case reports describing psychotic episodes associated with ayahuasca and DMT ingestion [32]. They found five case-based reports describing psychotic episodes associated with ayahuasca ingestion, and three case reports describing psychotic episodes associated with DMT. Most subjects, but not all, had a personal or family history of psychosis or mania. In Bremler et al.’s study 13–16% presented with overtly psychotic symptoms following their experience (e.g., highly unusual / magical ideas or auditory hallucinations) [27].

Zeifman et al. conducted a review on the relationship between classic psychedelics and suicidality suggesting that recent clinical trials provide no evidence for increased suicidality but instead point to acute and sustained decreases in suicidality following treatment [33]. However, McNamee et al. [12] point to evidence from trials using MDMA and psilocybin [22] showing increases of suicidal ideation and self-injury in over 7% of participants.

### Predictors of extended challenging experiences

Simonsson and colleagues’ aforementioned survey of diffcult and distressing experiences in psychedelic users looked at difficulties that extended beyond the psychedelic trip. They identified six set and setting variables as predictors of participants’ degree of difficulty of challenging experiences: no preparation, negative mindset, no psychological support, disagreeable social environment, dose too large, major life event prior to experience, and disagreeable physical environment [16]. Other factors identified included the use of lithium or other mood stabilizers.

The previously mentioned study by Carbonaro et al. suggests that the duration of the challenge is crucial in determining whether the experience is beneficial or harmful; the latter may be more likely with longer duration of difficult experiences [15]. Bremler and colleagues, based on their interview reports, highlight adverse contextual conditions and/or special psychological vulnerability (young age or psychiatric history) [27]. When feelings of increased sensitivity and vulnerability persist, they may lead to prolonged states of fear and anxiety. In Bremler’s study of challenging experiences, anxiety symptoms arose or worsened in 87% of participants.

In terms of setting variables that promote a positive outcome and limit adverse experiences, Johnson et al. [34] and Gorman et al. [35] conclude that preparation and taking psychedelics in controlled settings with a guide and clear knowledge of dose is vital to limiting the likelihood of acute adverse experiences. Bouso et al.’s study from the Global Ayahuasca Survey found that being female, unmarried, or having a pre-existing anxiety condition predicted adverse mental health outcomes after an ayahuasca ceremony, as did taking ayahuasca in a non-religious setting [17].

### The current study: Rationale, aims, research questions and hypotheses

The aim of the current study was to investigate difficult experiences persisting beyond 24 hours after the pharmacological effects of taking a psychedelic have concluded. It investigates the types, phenomenology, perceived causality and associated risk factors of these enduring difficulties. The study addresses a significant gap in the existing research landscape, particularly in terms of how such difficulties are described by those that experienced them. The below research questions and hypotheses are derived from the literature reviewed above, while extending it into novel areas.

### Quantitative research questions and hypotheses

The research questions that informed the descriptive quantitative data collection were as follows:

1. What types of difficulties are reported by individuals following a psychedelic experience, and what is the relative prevalence of these?
2. What contextual social settings and psychedelic substances precede the difficulties?
3. To what extent do participants attribute a relationship between enduring difficulties and (a) past traumatic experiences, or (b) prior or subsequent diagnoses of mental illness?
4. What are the current attitudes towards psychedelics among individuals who have previously encountered difficulties related to psychedelic use, and do they continue to use these substances?

In addition, quantitative hypotheses were formed to be tested by inferential statistical analysis. Based on findings and inferences from existing studies we also hypothesized that the following factors would predict a greater range of enduring difficulties: (1) No knowledge of dose by the participant or someone else present at the psychedelic trip, (2) Being in an unguided, supported setting at the time of the psychoactive experience, (3) A diagnosis of mental illness before episode, (4) Taking psychedelic substances known to have more intense effects [36], (5) A more challenging trip while the pharmacological effects of the drug were active. We also predicted that the same variables would predict a longer duration of difficulties.

#### Qualitative research question

The qualitative component of the study presented in the current article was directed by a single research question, which was as follows: How do individuals who experience difficulties after a psychedelic trip lasting more than 24 hours describe those difficulties in brief, written narrative form and what themes and patterns can be elicited from such data?

Responses to the quantitative and qualitative research questions are complementary in developing a better epidemiological and phenomenological understanding of extended adverse experiences. The analyses of both will be discussed in integrated form in the Discussion section.

## Methods

### Mixed-methods design

The study employed a convergent mixed-methods design, [37]. In a convergent design, quantitative and qualitative data are collected simultaneously about a phenomenon and then findings are presented together within a combined report, on the justification that they provide complementary information about the topic. The quantitative and qualitative elements are weighted equally in the study [a QUAN-QUAL design using Creswell and Plano Clark’s phrasing], rather than one being the main focus of analysis and the other being a secondary focus. They are then discussed as parts of a singular, multifaceted report. For the purpose of this study, we collected quantitative data and qualitative data through an online survey to gain key descriptive epidemiological categorical information and data for the purposes of statistical analysis about (a) the psychedelic experience itself, (b) the enduring difficulties after the usage of psychedelic drugs, and (c) perceived etiological factors, while also asking participants to describe the difficulties they experienced in a brief written narrative. These two forms of information about the topic of study are presented and analyzed within the results section and integrated further within the Discussion section below.

### Participants and recruitment strategy

There were three criteria for participation. These were (1) to have experienced difficulties after using a psychedelic drug, which negatively impacted functioning for more than a day after the psychedelic trip (there was no exclusion based on social context or purpose of taking the psychedelic), (2) be aged 18 or over, (3) write English to a proficient or fluent standard. Participants were recruited via a range of means: The online survey was distributed via multiple social media channels, via a newsletter about psychology and philosophy, via email lists to students, and via a newspaper advert. There were no financial incentives for participation. 608 individuals completed the survey between October 2022 and January 2023. All participants provided written consent for participation. Table 1 provides a breakdown of frequencies of demographic categories. The sampling strategy was randomized sampling, with no purposive stratifications, in light of the projected sampling size providing for representation of different demographic categories. The sample achieved provides satisfactory power for the quantitative analyses conducted [38].

**Table 1:** Frequencies of demographic categories within the dataset.

		Percentage of total sample
Gender	Female	49%
	Male	48%
	Other Gender	2%
	Not stated	1%
Age	Aged 18–24	11%
	Aged 25–34	29%
	Aged 35–44	26%
	Aged 45–54	19%
	Aged 55+	14%
	Age not stated	1%
Educational level	High school educated	15%
	Undergraduate degree	33%
	Masters degree	32%
	PhD or other doctoral degree	10%
	Other / prefer not to say	10%
Nationality	USA	32%
	British	22%
	Canadian	4%
	Other (49 countries represented in total, with each max 2% of sample)	41%
Ethnicity	White	84%
	Hispanic	3%
	Mixed ethnicity	5%
	Asian	2%
	Other	3%
	Not stated	3%

### Data collection

Ethical approval for the study from the University of Greenwich was gained prior to data collection commencing (application ref: 21.5.7.20). Data was collected anonymously via an online survey created in the online survey platform Qualtrics, between November 2022 and April 2023. The questionnaire comprised a written consent form, followed by a series of open-ended and closed-ended questions. Closed-ended questions about extended difficulties and interpretations of these are described in the Results section. Similar to Simonsson et al. [16], we measured how challenging the acute psychedelic experience was by way of a single item “How challenging was the psychedelic experience itself”, with a 4-point Likert scale response: Not at all challenging, Moderately challenging, Very challenging, Extremely challenging.

The variable: “Range of difficulties experienced” was calculated as the sum of difficulty types reported via the closed-ended question reported in Table 3, in which participants were presented with a set of extended difficulty categories (emotional, self-perception, cognitive, social, ontological, spiritual, perceptual, other) and ask to select all those that apply.

In terms of qualitative data collection, the instruction provided to participants to describe the difficulties they experienced in written form, was as follows: “Please describe the lasting difficulties that you encountered after your psychedelic experience. We would like you to write for about 3 to 5 minutes.” Participants were provided with a debrief form at the end of the questionnaire, which provided information about support organizations and information websites that help with psychedelic harm reduction and the integration process.

### Quantitative analyses

Given the number of tests conducted, the *p* value was corrected to 0.02 for significance using the Hochberg step-up method [39]. IBM SPSS v.28 was used to run all statistical tests. Parametric assumptions were met for regression and ANOVA analyses. Two regression analyses and one One-Way ANOVA were conducted to test the hypotheses pertaining to predictive risk factors for a longer duration of, and broader range of, extended difficulties.

### Qualitative analysis

The study employed Structured Tabular Thematic Analysis (ST-TA) to analyze the data [40]. This form of thematic analysis is designed specifically to analyze brief texts such as those elicited by open-ended questions in questionnaires. It uses spreadsheet software such as Excel to organize the data and thematizing. ST-TA draws on both the reflexive thematic analysis of Braun and Clark [41], and the ecumenical thematic analysis of Boyatzis [42]. It includes the calculation of theme frequencies as a means of conveying how common a theme is within a dataset. Frequencies are more meaningful in the kinds of brief-text datasets with higher participant numbers that can be processed using ST-TA, in contrast to the smaller samples typical to interview studies.

ST-TA can be conducted in inductive, deductive and hybrid forms. For the current study, given the relatively uncharted nature of the domain under study, particularly from a qualitative perspective, we chose to conduct an inductive analysis. The phases for inductive analysis in ST-TA are as follows: 1. Deep Immersion in the Data; 2. Generating Initial Codes and Themes; 3. Tabulating Themes Against Data Segments; 4. Checking Inter-analyst Agreement; 5. Exploring Theme Frequencies, 6. Developing thematic maps and diagrams, and 7. Producing the report [40].

With regards to Phase 4, ST-TA approach incorporates processes for establishing agreement between two or more analysts as part of ensuring that themes are transparently and cogently described and labelled, and that eventual conclusions are consensual and not based on idiosyncratic interpretations pertaining to an individual researcher. The process of reaching agreement aids in ensuring that theme names and descriptions are clear, non-overlapping and internally coherent.

ST-TA is cyclical, iterative and inter-subjective. Analysis proceeds through cycles of theme development and discussion with members of the research team to aim for consistency, rigor, transparency and inter-subjective agreement. A process within ST-TA that supports this is for the agreement-reaching process. Part of the this is calculating agreement levels via two analysts independently analyzing a subsample of texts, and aiming for 80% [40]. Using random samples of responses and analyst pairs, it took three iterations of agreement check to reach 80%, between which there were continued revisions of themes and sub-themes to boost clarity and cogency. This process of ensuring inter-subjective agreement helps to ensure a consensual, non-solipsistic approach to analysis within the conceptual framework of the study.

### Reflexivity and researcher characteristics

The design of this study was devised by an international, interdisciplinary team across multiple institutions, with specialisms in psychology, psychiatry, philosophy, and the humanities. Analysis of the data was conducted by three researchers: an interdisciplinary academic specializing in the intersection of philosophy and psychology (Analyst 1), a psychologist specializing in qualitative methods and mixed methods (Analyst 2), and a psychology researcher specializing in psychedelics (Analyst 3). The interactive dialogical approach was taken to data analysis, which involves a series of mutually influential interactions between a group of analysts [43]. There were eight scheduled discussions between the members of the team during the analysis process, in which interpersonal and interpersonal reflexive deliberations and possible sources of bias were discussed with the aim of maximizing transparency and accuracy. This process led to the synergistic development of a transferable, parsimonious, and accurate thematic scheme that was a synergistic product of the knowledge basis and analytical abilities of all three individuals.

## Results

### Quantitative descriptive results

#### The psychedelic experience: Substance taken, dose knowledge, location

Participants reported what substance they had taken during the experience that led to the enduring difficulties. They could report more than one substance if appropriate. The two most commonly reported were psilocybin (27%) and LSD (25%), followed by ayahuasca (10%), cannabis (10%), MDMA (7%), DMT (5%), ketamine (4%), mescaline (2%) and *salvia divinorum* (1%). 8% selected Other and could specify in text. Of these others, iboga, 5-MEO-DMT, PCP, Bufo (*Incilius alvarius* toad venom), 2C-B, 2C-E, nitrous oxide, and sananga were mentioned. Non-psychedelic drugs that were also mentioned in addition to the psychedelic were methamphetamine and cocaine.

Participants were asked if they or someone else present knew the dose being given. 449 (73.8%) responded yes. 156 (25.7%) responded no, and 3 did not give a response.

Table 2 shows the frequency and percentage of the sample for the stated locations where the psychedelic experience was reported to have occurred. The most common was “with a friend, partner or group of friends”, followed by “on my own”, and “in a group ceremony” which was the third most common. These location types are grouped into guided (settings with a psychedelic guide, shaman or specialist leading the experience) and unguided (settings without a specialist guide) for later analysis purposes.

**Table 2:** The location of the psychedelic experience with frequencies and percentages.

Social Setting	Type	Frequency	Percentage
With a friend, partner, or group of friends	Unguided	213	35.0
On my own	Unguided	115	18.9
In a group ceremony	Guided	73	12.0
At a rave, nightclub or festival	Unguided	57	9.4
Other	Unguided	42	6.9
On a psychedelic retreat	Guided	39	6.4
At a psychedelic therapy session	Guided	32	5.3
At a party	Unguided	18	3.0
At a clinic or medical trial	Guided	17	2.8
Information not provided	N.A.	2	0.3

#### Post-experience difficulty duration and types

**Fig 1:**
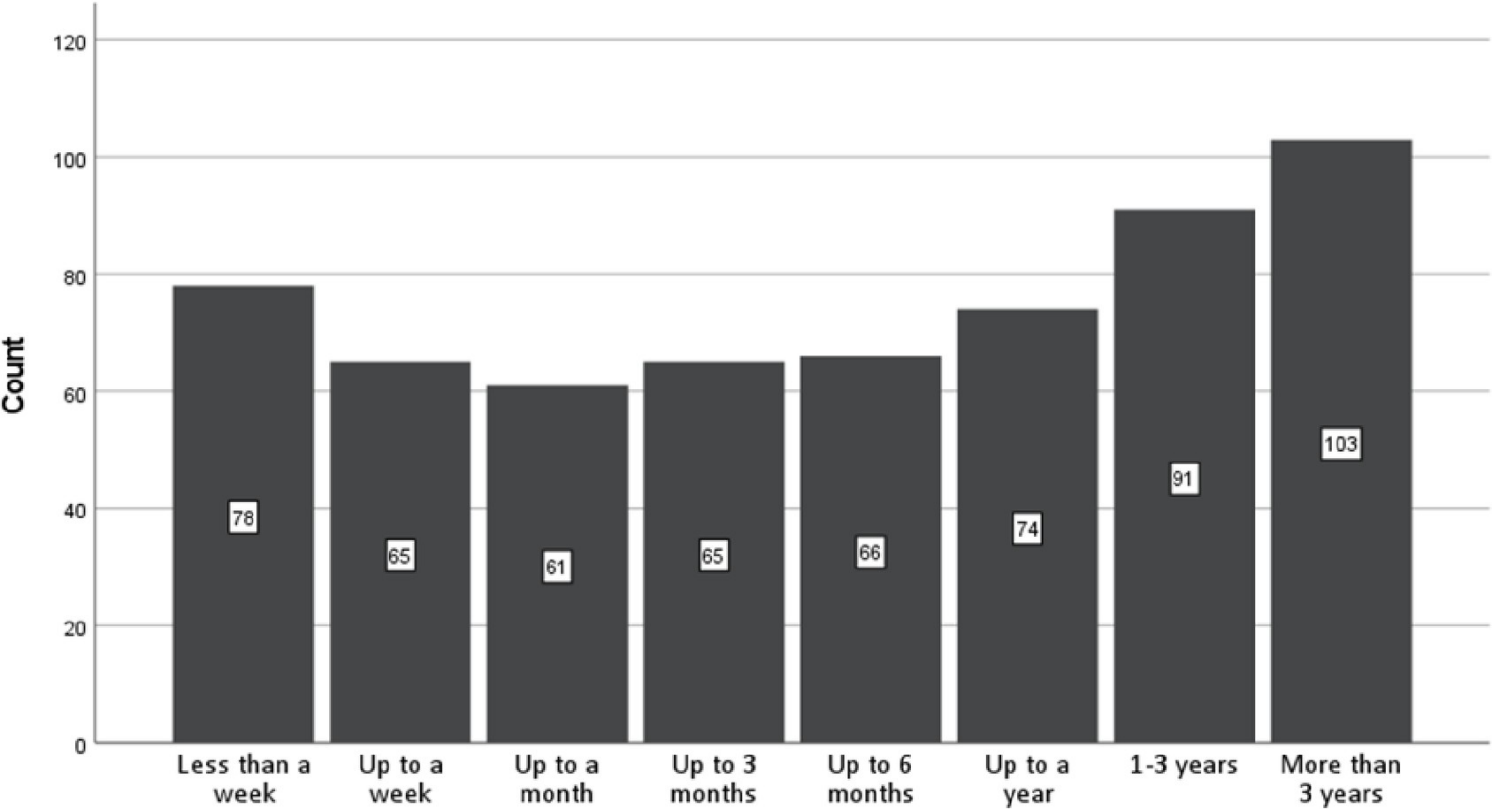
The duration of the difficulties after the psychedelic experience: Response frequencies for duration categories provided.

Fig 1 shows the frequencies of responses to the duration categories provided to indicate how long the difficulties after the experience lasted. The most frequently endorsed categories were 1–3 years and >3 years, showing a high prevalence of extended difficulties. There is also a visibly curvilinear trend such that there are peaks with less than a week and over a year, with a dip around a month.

Table 3 shows data frequencies and percentages of the sample from responses to a close-ended question about what types of difficulties participants experienced. The full wording provided to participants is shown in the table. Participants could select multiple responses if needed. The most endorsed difficulty type was emotional difficulties, with 76% of participants reporting these, followed by self-perception difficulties (58%), and cognitive difficulties (52%). It is of note that 50% of the sample experienced ontological difficulties and 34% reported spiritual difficulties. These types are explored further in the thematic analysis.

**Table 3:** Difficulty types (from closed-ended list) reported after the experience ended for at least one day, with frequencies and percentages.

Difficulty Type	Frequency	Percentage
Emotional difficulties (e.g., the way you felt emotionally or the ability you had to emotionally regulate)	464	76
Self-perception difficulties (e.g., the way you felt about or understood yourself)	355	58
Cognitive difficulties (e.g., the way you thought about things)	318	52
Social difficulties (e.g., the way you interacted the related to other people)	316	52
Ontological difficulties (e.g., the way you understood reality and existence)	304	50
Spiritual difficulties (e.g., your spiritual beliefs)	209	34
Perceptual difficulties (e.g., the way your vision or hearing functioned)	156	26
Other Difficulties	125	21

#### Participant etiological interpretations: Mental Illness, childhood trauma

Participants were asked if they had been diagnosed with a mental illness prior to the experience. 173 (28.5%) responded yes, while 410 (67.4%) responded no. 25 did not respond. Those participants who answered yes were then asked “Do you think this prior diagnosis may be linked to the difficulties you experienced after the psychedelic experience?”. To this follow up question, 79 (45.9%) responded yes, while 50 (29.1%) responded no. 43 (25%) responded not sure.

Participants were asked: “Was there a traumatic experience in your childhood or youth which you think may have played a role in the difficulties that arose during or after the psychedelic experience?”. 243 (40%) responded yes. 180 (29.6%) responded no, and 158 (26%) responded not sure. 27 participants did not provide an answer.

Participants were then asked about mental illness onset subsequent to the psychedelic experience in question. In response to the question “Have you been diagnosed with a mental illness since the psychedelic experience?” 114 (18.8%) said yes, while 467 (76.8%) said no. Those who responded yes to that question were then asked “Do you personally think that your psychedelic experience contributed towards this diagnosis?”. 59 (53.6% of those with a post-experience diagnosis) responded Yes; 25 (22.7%) responded No; and 26 (23.6%) responded not sure.

#### Continued consumption of, and attitudes to, psychedelic drugs

**Fig 2:**
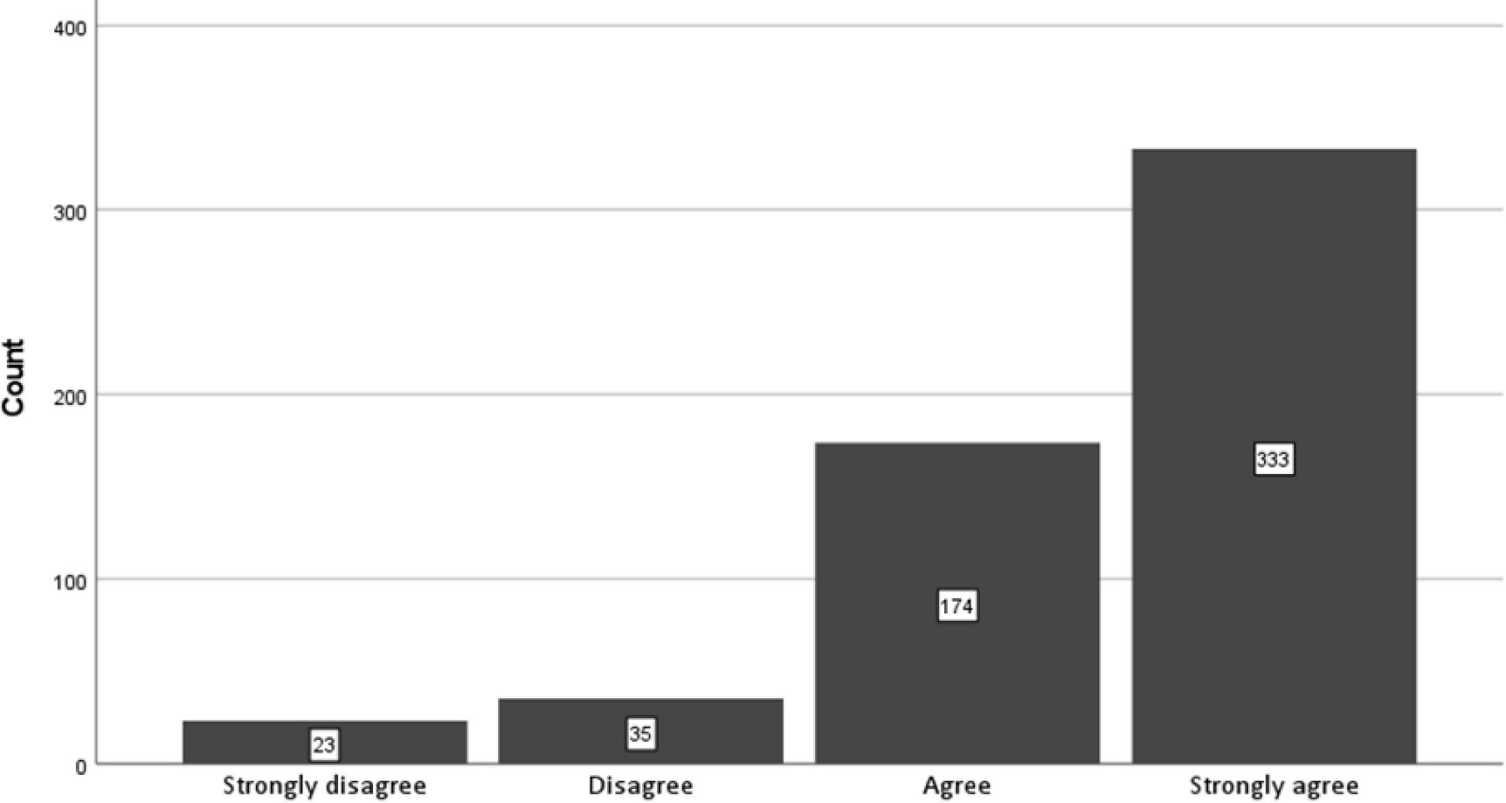
Response frequencies to the question “I believe that the insights and healings gained from psychedelics, when taken in a supportive setting, are worth the risks involved”.

Participants were asked if they still take psychedelic drugs. In response, 334 (54.9%) said yes, and 246 (40.5%) said no. 28 did not respond to the question. They were then asked to rate their agreement with the following statement: “*I believe that the insights and healings gained from psychedelics*, *when taken in a supportive setting*, *are worth the risks involved*.” The frequencies across the four response points to this question (Strongly Agree, Agree, Disagree, Strongly Disagree) are shown in Fig 2. In total, 89.7% agreed or strongly agreed with the statement.

#### Hypothesis testing: Predictors of range and duration of difficulties

We hypothesized that having no knowledge of dose, being in an unguided setting, having diagnosis of prior mental illness and the challengingness of the psychedelic trip itself would predict range and duration of extended difficulties. To test this, linear regression analyses were conducted, firstly with range of difficulties as the DV and secondly with duration of difficulties as the DV. The overall regression model for the prediction of range of duration was significant (R^2^ = .04, F(4,519) = 4.97, p < .001). In terms of individual predictors, two emerged as significant; the challengingness of the trip (β = .17, p<0.001) and being in an unguided setting (β = .11, p = 0.008).

The overall regression model for the prediction of difficulties duration was significant (R^2^ = .06, F(4,518) = 7.73, p < .001). In terms of individual predictors, the challengingness of the trip (β = .21, p<0.001) emerged as significant within the model. Being in an uncontrolled setting and not having a knowledge of the dose were in the predicted direction but not significant.

We also predicted that duration and range of difficulties would differ by psychedelic drug taken. Participants who reported more than one drug being taken together were removed, and two groups were removed that did not meet the minimum requirements for group size (k+1), which were *salvia divinorum* and mescaline. Then two One-Way ANOVAs were run, with drug as IV, and (a) duration of difficulties, and (b) range of difficulties as DVs. Drug type exerted a significant effect on duration F (9,477) = 2.89, *p* = 0.002, but not on range, F (9.480) = 1.33, *p* = 0.06. Post-hoc LSD tests established that the effect on duration of effects was principally driven by significant differences between (a) ayahuasca, DMT and LSD as high values and (b) psilocybin and MDMA as low values.

### Qualitative analysis of the written narratives of extended difficulties

590 participants provided written accounts of the difficulties they experienced. Table 4 shows main themes, subthemes, frequencies and percentages of reports evidencing the subthemes that were elicited through the inductive thematic analysis. The main themes show similarities to the difficulty types elicited via the closed-ended question but were generated inductively from subthemes. We found that ontological and spiritual difficulties were sufficiently overlapping to combine into a single main theme.

**Table 4:** Thematic analysis main themes with frequencies and percentage of total sample, and subthemes with frequencies and percentage of total sample.

Main theme	Main theme frequency and %	Subtheme	Subtheme frequency and %
1. Social Difficulties	164 (27%)	Sense of disconnection from others / society	75 (13%)
		Communication difficulties	38 (6%)
		Social anxiety / fear of ostracism	31 (5%)
		Hurt by behavior of others during / after experience	27 (5%)
		Social withdrawal / social shut down	14 (2%)
		Sense of stigma about the experience	12 (2%)
		Difficulty with being socially ’normal’	11 (2%)
2. Perceptual Difficulties	125 (21%)	Visual hallucinations / visual disturbance	71 (12%)
		Flashbacks / feeling of experience being repeated	44 (7%)
		Non-specific sensory disturbance / hallucinations	15 (3%)
		Auditory distortions / hallucinations	13 (2%)
		Time distortions	6 (1%)
3. Cognitive Difficulties	110 (18%)	Difficulty with thinking clearly / confusion	53 (9%)
		Intrusive / ruminative / obsessive / fixated thoughts	40 (7%)
		Forgetfulness / memory issues	13 (2%)
		Difficulty with concentration and focusing	13 (2%)
		Difficulty making decisions	9 (2%)
4. Emotional Difficulties	406 (67%)	Anxiety, fear andworry (not specific to an object or person)	153 (26%)
		Depression	68 (12%)
		Paranoia / problems with trust	66 (11%)
		Panic attacks	52 (9%)
		Low mood / bad mood / sadness / anhedonia	49 (8%)
		Challenging / fluctuating / overwhelming emotion	40 (7%)
		Shame / guilt	39 (7%)
		Fear of going mad or insane	38 (6%)
		Suicidality / self-harm thoughts	37 (6%)
		Fear of damage / permanent damage to brain or self	36 (6%)
		Anger / frustration / irritability	35 (6%)
		Resurfaced trauma	34 (6%)
		Feelings of vulnerability and fragility / feeling unsafe	34 (6%)
		Fear of death (not fear of afterlife, fear of hell)	33 (6%)
		Hyperarousal / hypersensitivity / hypervigilance	31 (5%)
		Traumatized by trip or what happened after	30 (5%)
		Phobias and fears of specific events or objects	27 (5%)
		Fear of the experience repeating / being spiked	17 (3%)
		Disappointment relating to experience and outcome	8 (1%)
		Fear of being alone due to state of mind	8 (1%)
5. Existential / Ontological Difficulties	253 (42%)	Existential Struggle	102 (17%)
		Derealization	86 (15%)
		Struggle to integrate experience into everyday life	58 (10%)
		Magical / irrational / delusional beliefs	38 (6%)
		Sense of hell / evil presence / fear of afterlife	24 (4%)
		Hostile / impersonal reality / nihilistic sense	19 (3%)
		Despair over state of the world / other people	15 (3%)
		Experience of possession/curse	14 (2%)
6. Self-Perception Difficulties	143 (23%)	Depersonalization / dissociation	95 (16%)
		Diminished or disempowered self	51 (9%)
		Excessive self-consciousness or self-awareness	9 (2%)
7. Somatic Difficulties	116 (19%)	Sleep problems and nightmares	54 (9%)
		Non-specific somatic issue	22 (4%)
		Fatigue	18 (3%)
		Headaches	15 (3%)
		Crying (compulsive, extended)	14 (2%)
		Compulsive movements or actions	9 (2%)
		Weight loss / gain	8 (1%)
		Increased sensitivity to other drugs	8 (1%)
		Breathing problems	7 (1%)
		Heart issues / chest pains or chest sensations	7 (1%)
		Nausea	5 (1%)
8. Behavioral Difficulties	65 (11%)	Difficulty with performing in career / studying	36 (6%)
		Substance use / abuse	12 (2%)
Experience of Psychosis	29 (5%)	Sense of going through a psychotic episode	29 (5%)

### Social difficulties

The first main theme was *Social Difficulties*, which were reported by 27% of respondents. Within this theme, the most commonly reported type of social difficulty was *Sense of Social Disconnection*, which subsumes comments about being ‘isolated’, ‘lonely’ and ‘disconnected from others’. This was reported by 13% of our respondents, in quotes such as the following:

*A feeling of being distant, withdrawn*, ’*socially outcast*’… *Even from other Ayahuasca users*.

Further sub-themes within Social Difficulties included *Communication difficulties*–difficulties talking to others or understanding them; *Sense of social stigma about the experience*–feeling ashamed of your experience and hence not talking about it; *Social anxiety*–feeling a heightened fear of others’ judging you; feeling *Hurt by the behavior of others*–for example by the behavior of the guide during a ceremony. This behavior could be during or after the trip, although we only coded it if the respondent’s hurt feelings continued beyond the trip itself. There was also *Social withdrawal or shut down*–a more extreme form of social disconnection, for example

*I became withdrawn*, *untrusting*, *disengaged from friends*. *I recall giving away my possessions*. *Though smart*, *I dropped out of high school*.

Finally, *Difficulty with being socially normal*–where people describe wanting to fit in and be ‘normal’ but no longer knowing how to do that.

### Perceptual difficulties

The second main theme was *Perceptual Difficulties*, reported by 21% of participants. The most common sub-theme in this theme was *Visual distortions / hallucinations*, which was reported by 12% of respondents. This sub-theme could include phenomena akin to Hallucinogen Persistent Perception Disorder (HPPD), for example people describing continued visual snow syndrome, visual perceptual distorting, seeing tracers, and extreme sensitivity to light. For example:

*Multiple times throughout the day if I focus on something*, *my vision looks like it’s stretching and moving around gets worse the more I try to look for it*.

There were also five reports of prosopagnosia (difficulty recognizing faces) which we included in the *Visual distortions / hallucinations* sub-theme. We coded *Flashback* as a separate sub-theme in this category, when people simply wrote ‘flashback’ or described feeling like the trip was repeating, or reported that they woke up tripping in the nights after the trip. Sometimes this sounded like a traumatic response, as in:

*It was absolutely traumatic*. *I have PTSD from it and experience flashbacks of the trip*.

It’s an interesting question whether this sort of post-traumatic ‘flashback’ is a different phenomenon to HPPD-type extended visual distortions. If people wrote both ‘HPPD’ and ‘flashbacks’ we coded as both *Visual distortions / hallucinations* and *Flashbacks*.

Also in the *Perceptual Difficulties* theme is the sub-theme *Auditory distortions / hallucinations*–this includes both increased sensitivity to noise, auditory distortions like buzzing, and more extreme auditory hallucinations like hearing voices. In the sub-theme *Non-specific sensory distortions / hallucinations* we included tactile, taste and olfactory distortions or hallucinations that continued beyond the psychedelic experience. In *Time distortions* we included people reporting extended distortions to their sense of time, as in this example:

*Time did not feel the same for up to a week afterwards*, *everything felt like it was moving a lot slower than it should’ve and this caused me to think i was going crazy leading to stress and low mood*.

### Cognitive difficulties

18% of survey respondents reported *Cognitive Difficulties* after a psychedelic experience. The most common sub-theme in this theme was *Confusion / problems with thinking clearly*–this sub-theme, which turned up in 9% of survey responses, related to immediate feelings of confusion rather than more abstract existential confusion over the meaning of life. Here’s one example:

*I couldn’t think or speak linearly for at least a few days*, *or at least it felt that way*.. *Everything felt confusing and I didn’t know if or when it was going to end*…*I didn’t understand what was happening to me*, *and did not have any language for it*.

Another sub-theme in this category is reports of *Intrusive*, *ruminative or obsessive thoughts*, such as:

*After my trip was over I felt shaken up for days/weeks and wondered if I had gone crazy*. *Thoughts like this were going around in my head during those weeks*: *"What really IS ’crazy’*?*" "Whatever the majority thinks is not suitable behavior*?*" "Am I ’crazy’ for not feeling that I want to adapt to this world*?*" "Isn’t it a crazy thing to be well-adapted to a crazy world*?*" "Isn’t everyone ELSE crazy then*?*" "Crap*, *that’s such a crazy-person thing to say*!" *Round and round it went*.

Another Cognitive Difficulties sub-theme is *Forgetfulness / memory issues*, and the last two sub-themes in this category are *Difficulty with concentration* and *Difficulty with making decisions*.

### Emotional difficulties

The most common type of extended difficulties that people reported were *Emotional Difficulties*, which affected 67% of survey respondents. Within this theme, reports coded as *Anxiety / fear* were the most common sub-theme, with 26% of all responses mentioning these words, such as:

*For about 18 months*, *I awoke with the sun every morning full of a feeling of absolute terror*…*Sometimes my anxiety would be so high in the morning that i would physically shake from the energy*.

Many varieties of anxiety and fear were also reported and coded as separate sub-themes–*Paranoia* by 11% of respondents, *Panic attacks* by 9%, *Social anxiety* by 5% (included in the Social Difficulties category), and also reports of *Fear of the experience repeating* (a theme for which we included fear of being spiked), *Fear of losing control or going mad*, *Fear of being alone’* (usually because people were afraid to be alone with their mind), *Other forms of phobia* such as agoraphobia, *Fear of dying* (especially common if respondents felt they were dying during the trip), and *Fear of permanent damage*, for which we coded statements such as: ‘I felt like my brain was broken and would never feel normal again’. Another 6% reported *Feeling vulnerable or unsafe in their own mind* after the trip, as in this example:

*I believe the experience contributed to my general anxiety for a time*, *as I became aware that my psyche was not necessarily a* ’*safe’ place to exist within*, *and possibly played a role in my fear of being alone*, *which I suffered with around that time of my life*.

Other Emotional Difficulties sub-themes included *Depression*, which was reported by 12% of respondents, *Low mood / anhedonia*, *Challenging or overwhelming emotion*, *Anger / irritability*, *Hyperarousal / feeling unable to relax / hyper-vigilance or emotional sensitivity’*, and *Suicidality*, which was reported by 6% (including reports by family members of two people who killed themselves after bad trips). Here’s a response we coded for *Depression* and *Suicidality*:

*I collapsed into a severe*, *almost catatonic depression*. *I could not even tell my husband that I was having suicidal ideation*. *I reached out to my therapist for integration sessions once and when he did not reply*, *I fell further into the abyss of hopelessness and despair*. *I went through the motions of existing*, *grateful that my busy schedule held me accountable for staying alive*, *but I did not see the point*. *This lasted over 2 months*.

People sometimes emerged from bad trips with a deep feeling of guilt or shame, which we coded under the sub-theme *Guilt / shame*:

*I feel like somehow I failed because I did not get favorable result and feel shame in discussing it*. *I think this shame comes from the fact that I shut down and did not let medicine work*. *This in turn makes me feel a bit hopeless or too difficult a case for medicine but I know this is just my insecurities and fundamental belief that I am not worthy*.

Another theme in this category is *Resurfaced trauma*, where people felt the psychedelic experience had released buried trauma which they then felt overwhelmed by, as in this example (also coded for *Flashbacks*, *Guilt / Shame* and *Diminished Sense of Self)*:

*Had a traumatic memory surface and I was on my own and it was extremely hard to navigate*. *Afterwards I am struggling with making meaning*, *continue to have trauma flashbacks and feel so terrible about myself*. *My self-esteem feels worse and I don’t know how to get a handle on my intrusive thoughts and ways I am now thinking about myself*. *Feel ashamed of who I am and the decisions I’ve made*. *Feel like my life has been made worse by what happened and I’m powerless to change it*.

Finally, within the *Emotional Difficulties* category we included a sub-theme for *Feelings of disappointment after the trip*, for eight reports where people struggled with the fact that the ‘miracle cure’ they hoped for from psychedelics did not occur.

### Existential and ontological difficulties

In the *Existential and Ontological Difficulties* theme, which included 42% of survey responses, we included sub-themes reporting varieties of existential, spiritual, and ontological difficulties, involving struggles around meaning and the nature of reality. The most common sub-theme in this theme was *Existential struggle*. In this sub-theme we included any reports which expressed existential confusion, or struggles to make sense or meaning of psychedelic experiences or reality in general following a trip. Around 17% of respondents expressed some form of existential struggle. Examples include:

*I entered the experience believing that my experience is the literal real external world*. *The experience contained me living out my worst fears*, *the deepest possible shame*. *Other experiences so bizarre and dreamlike I could not make sense of them*. *These memories left a legacy of confusion about what deeper model of reality to use*, *and repeated experiences of flipping between these models at different times*.

15% of responses were coded for the sub-theme of *Derealization*, in which people expressed confusion or uncertainty over what was real in the days, weeks or months after a psychedelic experience, sometimes feeling they were in a dream, afterlife, purgatory, a movie, a computer game or fake reality. This sub-theme was often accompanied by reports of *Depersonalization*, which we coded under the category of *Sense-of-Self Problems*. Here is one example of *Derealization*:

*almost exactly 2 months after the trip*, *something happened*. *I was in a restaurant and all of a sudden I began to feel like something was wrong*. *I went to the bathroom*. *All of a sudden*, *the bathroom was not real*. *It just looked WRONG*. *I had to get out of there*. *But when I left the restaurant*, *the street outside was not real either*. *The whole world was simply not real*, *and I felt like I had to vomit*.

Other sub-themes in this category include *Sense of hostile / impersonal reality*, in which people’s attitude to the universe shifts to a negative sense of the universe as an unfriendly place; and *Sense of hell / evil presence / fear of the afterlife*, in which we included responses where people felt like they were stuck in a hellish space during their trip, or that they had encountered demonic beings, and they subsequently become terrified of re-encountering hell or demons after they die. Under the *Possession* sub-theme we included reports where people felt they had been possessed by a spirit or energy, or by another person. Under *Magical / delusional beliefs* we included responses where people’s sense of agency felt supernaturally expanded, so that they could magically control other people, events or destiny–and they felt that this was in some way an unpleasant or anxiety-inducing situation. Under *Despair over the state of the world or the human race*, we included reports where this despair was experienced as a difficulty that obstructed people’s day-to-day functioning. Finally, we included a sub-theme *Struggle to integrate the experience and return to daily life*.

### Self-perception difficulties

The theme of *Self-Perception Difficulties* included any difficulties related to changes in people’s sense of self and identity. The most common sub-theme in this category was *De-personalization*, with 16% of survey responses being included in this sub-theme. We coded this if people explicitly reported suffering from depersonalization, loss of self (in a way that felt unpleasant and difficult to manage), fragmented, dissociated, disconnected or untethered from their former self or from their body. For example:

*I felt like the person I was before had been entirely wiped from all sense memory and I felt completely dissociated from the body I was inhabiting*…*[I] essentially felt like I was completely disintegrating*. *My life has and never will be the same*.

Within *Diminished or disempowered self* we included responses where people reported feeling worse about themselves, lower self-esteem, less confident, competent or empowered. For example:

*I felt really down afterwards*, *disempowered within myself*, *unsure and uncertain of myself*, *lack of confidence*.

Finally, under the sub-theme *Excessive self-consciousness or self-awareness* we included responses where people felt affected by heightened self-consciousness.

### Somatic difficulties

Under the theme of *Somatic Difficulties* (reported by 19% of the sample), we included any problems related to the body, such as *Breathing problems*, *Heart issues* or *Nausea*. The most common sub-theme in this category was *Sleep problems*, *including nightmares*, which affected 9% of survey respondents. Usually this took the form of nightmares after bad trips, or people waking up feeling like they were still tripping in the nights after a trip, which led to a fear of going to sleep. Other sub-themes in this category include *Weight loss / gain / eating problems*, *Headaches / dizziness*, *Compulsive movements* such as twitching, trembling or even screaming, *Compulsive crying*, *Non-specific or other somatic issues* (for example muscle spasms or fevers), and *Increased sensitivity to other drugs*.

### Behavioral difficulties

In the *Behavioral Difficulties* theme, we included two sub-themes connected to people’s behavior. Firstly, *Substance abuse problems*– 2% of respondents said they developed substance abuse problems following a difficult psychedelic experience. And 5% of respondents said they had experienced *Difficulties with performing their work*, *career*, *studying*, *or managing their finances* after a psychedelic experience.

### Experiences of psychotic episode

We included reports of a psychotic episode as a stand-alone category, given that it did not fit within any of the above main themes but rather straddles across many of them. We included responses in this theme if people expressly wrote they felt psychotic or were diagnosed with a psychotic disorder after a psychedelic experience. For example:

*I stayed up for 3 days after dosing and went into psychosis*, *I believed all my friends were ‘spies’ talking to me to make fun of me*. *I was also throwing stuff at the wall and talking about wanting to kill myself on Day 3*. *This belief persisted until I’d been on an antipsychotic for months*.

## Discussion

The aim of this study was to investigate the enduring adverse experiences following the use of psychedelic drugs. We looked at the phenomenology, perceived etiology, relationship to risk factors, and relationship to continued usage/attitudes to psychedelics of such difficulties. Our first quantitative research question looked at the prevalence of types of these difficulties. When provided with a fixed checklist of emotional, self-perception, cognitive, social, ontological, spiritual and perceptual difficulties, emotional difficulties emerged as the most prevalent, and perceptual difficulties as the least prevalent. The qualitative research question looked at the nature of the difficulties within the brief written narratives. When describing their difficulties in this way, and with coding these inductively into meta-themes and subthemes, the most prevalent meta-theme was Emotional Difficulties, supporting the quantitative checklist, with “*Anxiety*, *fear and worry (non-specific)”* being the most prevalent subtheme, with a suite of other subthemes that also relate to fear and anxiety, such as paranoia, panic attacks, fear of death, feelings of being unsafe, hypervigilance and phobias. In summary, what stands out from both quantitative and qualitative analysis is that emotional difficulties are the most common form of enduring difficulties, and these most commonly take the form of anxiety, fear and panic. We discuss implications of this towards the end of this section. A further conclusion that emerges from the quantitative and qualitative findings together is that extended difficulties are highly heterogenous in duration, intensity and phenomenology, and this internal differentiation requires targeted studies to better understanding, for example, difficulties that last more than a year or specific kinds of challenges such as ontological difficulties.

Our findings supports the results of Simonsson et al., who found that anxiety was the most common enduring difficulty, based on quantitative questionnaire data [16] and Bouso et al’s study of the Global Ayahuasca Survey, in which ‘feeling nervous, anxious or on edge’ was the second most common adverse mental health effect [17]. Our findings also suggest that a *Sense of disconnection from others* was within the top five most prevalent themes, as did the studies by Simonsson et al. [16] and Bouso et al. [17]. Some extended adverse effects that were quite common in other studies weren’t so common in our data set–for example, feeling a harmful connection to the spirit world was reported by 14% of respondents to the Global Ayahuasca Survey but by less than 4% of our data set, which may suggest some forms of difficulty are particularly associated with certain psychedelic substances and/or their associated cultures [17].

The second quantitative research question asked what contextual social settings and psychedelic substances precede extended difficulties. The most common social contexts for the psychedelic experiences that preceded the extended difficulties were ‘with a friend, partner, or group of friends’ or ‘on my own’. When comparing social settings in this dataset (which is of course exclusively from individuals who reported extended difficulties), to social settings reported in a sample of general psychedelic usage [44], 12% of the current sample reported taking it at a party or similar event, but only 3% did in the comparison sample of all users, and while 18.9% our of our sample reported taking in in solitude, 43% of the general sample did. This suggests an avenue for future enquiry could be in looking at specific social contexts as risk factors for extended difficulties. 8% of our respondents experienced extended difficulties after taking part in a clinical trial or psychedelic therapy session. Thus, our data suggests that taking psychedelics in a clinical setting is not risk-free [45].The psychedelics most frequently mentioned as preceding extended difficulties were psilocybin and LSD, however this may simply reflect higher prevalence use. Ayahuasca, DMT and LSD were associated with the longest average duration of difficulty. Carbonaro and colleagues’ previous research suggested the duration of the difficult experience was predictive of adverse outcomes and they recommend that therapeutic interventions aim at reducing the duration of the challenge [15]. In the current study, 40% of the sample stated that they thought a childhood trauma was implicated in experiencing the post-psychedelic difficulties. This has similar theoretical connotations to the Simonsson et al. finding that a major life event prior to the experience was predictive of degree of enduring difficulty, in terms of antecedent life events being formative to psychedelic outcomes [16]. Previous research has identified a strong link between childhood trauma and developing dissociative symptoms following classic psychedelic use [46] as well as increased vulnerability to the development of psychiatric disorders [47].

Our third quantitative descriptive research questions focused on the extent to which participants attribute a relationship between enduring difficulties and (a) childhood traumatic experiences, or (b) prior or subsequent diagnoses of mental illness. These questions were answered, with figures for both being just under half of participants. We will be investigating these perceived etiological links within interview-focused studies in subsequent studies.

Our fourth quantitative research question looked at current attitudes towards and usage of psychedelics among this sample of individuals who have previously encountered difficulties related to psychedelic. The majority reported continued usage, and almost 90% report a positive view on the therapeutic benefits of psychedelics. This may point to the formative role of difficult experiences within the integration process. Bathje et al. state that overcoming difficulties may actually catalyze the integration process for some individuals [48]. There is also empirical evidence that trips involving major personality changes like the overcoming of addiction or the confrontation with repressed trauma are, unsurprisingly, challenging but nonetheless ultimately experienced as healing [49, 50].

Whether a phenomenon is experienced as ‘bad’ or ‘part of the process’ depends on a person’s appraisal. As with meditation experiences, emergent phenomena like depersonalization could be appraised as expected and welcome by some individuals, while others may find them unexpected and unsettling. Indeed, the same person might appraise such phenomena differently at different life stages. Some individuals might intentionally use psychedelics as a tool to revisit traumatic memories, while others in our survey reported feeling overwhelmed by the resurfacing of traumatic experiences. As previous work has cautioned, re-triggering trauma with psychedelics can augment maladaptive processes and associated defense mechanisms, especially in individuals with increased vulnerability due to childhood trauma [47].

Previous research supports the notion that challenging extended psychedelic experiences are not necessarily adverse outcomes. In Simonsson’s 2023 study, 78.6% of participants reported they were glad they had used psychedelics, after reflecting on their most difficult experiences [16]. A third of the participants interviewed by Bremler et al. attributed personal growth to the difficulties triggered by their psychedelic experience [27]. Gashi et al. also previously showed that ‘bad trips’ can be narratively transformed into valuable experiences [51]. 84% in Carbonaro’s study reported they benefited from their challenging trip even when it was one of the most difficult experiences of their lives [18]. Related to this, the same study found the degree of difficulty of an experience correlated positively with the degree of personal meaning gained from the experience. Also, 67% participants in the Johnstad (2021) study reported long-term consequences of their worst psychedelic experience to be positive, while only 4% reported negative consequences [13]. Bouso et al. found that 55% of respondents to the Global Ayahuasca Survey reported adverse mental health effects, but 88% thought such effects were ‘part of a positive process of growth or integration’ [17]. In the 2022 Canadian Psychedelic Survey, 56% of those who had an intense challenging experience reported that ‘some good’ came from the experience [52].

Providing some support for these previous findings, in the current study, 55% continue to take psychedelics to the present day, and 90% agreed that psychedelics can be helpful and are worth the risks if taken in supportive settings. Nonetheless, almost half no longer take psychedelic drugs, and in some instances, respondents reported feeling significantly harmed by their psychedelic experience and expressed regret over ever trying these substances.

In terms of the hypotheses that were tested, these were partially supported. Experiencing a greater range of difficulties was predicted by being in an unguided setting at the time of the trip and having a more challenging trip. Duration of difficulties was predicted by the challengingness of the trip but no other factors emerged as significant. We also found that difficulties following the use of ayahuasca, DMT and LSD were longer than other drugs. A prior diagnosis of mental illness did not predict the duration or variety of enduring difficulties. This supports the findings from Simonsson et al. that a high dose (which is likely in the absence of clear knowledge of dose) and a disagreeable physical environment were predictive of degree of difficulty [16].

### Limitations and future research

This study has contributed some of the first insights within published literature regarding the nature and predictors of long-term difficulties following psychedelic experiences, based on a large, multinational sample. Recruitment was achieved by a range of different means, and despite the size and multifaceted heterogeneity of the sample, our survey respondents were mainly Western and English-speaking, and mainly white. The generalizability of our findings remains an open question. Would the same kinds of difficulties and risk factors manifest in a country where psychedelics are legal? Or subcultural contexts such as the Santo Daime religion or the Native American church, where specific psychedelics are used as sacraments in religious ceremonies? An important next step is to examine whether different ethnic and cultural groups experience different sorts of psychedelic harms or are helped by different sorts of coping techniques, and if bad trips and extended difficulties are less common in cultures with a more established cultural relationship to psychedelic experiences.

In terms of the survey data collection type, written narrative data about difficulties was used alongside the closed-ended questions. Such brief written narratives have the advantage of being anonymous and they can be written in the participants’ own time, so allow for flexible data completion. They are however inevitably brief accounts of difficulties, and their spontaneous recalled nature means they are not comprehensive accounts. Unlike in interviews, there is no opportunity for follow-up questioning or probing. Building on these findings, the subsequent phase of our research project, focusing on challenging post-psychedelic experiences, will entail a series of studies involving in-depth interviews with specifically selected groups of participants. The aim is to acquire more detailed phenomenological data and a more contextualized understanding of these difficulties. For instance, 40% of respondents reported experiencing childhood trauma that they believed could be linked to their post-psychedelic difficulties. A follow-up study will be devoted to exploring this aspect. An additional study will specifically target individuals who reported experiencing an ’existential struggle’ following a psychedelic experience.

The data gathered in this study consists of self-reported recollections of individual post-psychedelic difficulties, which are influenced by memory and personal interpretative understandings of the event and its causes. Therefore, we refrain from inferring any causal relationships from this data. Our aim is to provide insight into the personal realities of those who recall such difficulties, in the hope that this will inspire further research studies equipped to make causal inferences. There may be individuals who are not willing to convey their difficulties, or who don’t interpret their experience as adverse but those around them do. And there may be groups whose experiences are not captured by self-reporting online surveys, such as those experiencing chronic mental health difficulties.

We did not give participants the option of stating how long each difficulty type lasted nor of the relevant valence or impact of the difficulty types. Future research could incorporate questions related to the experienced duration, significance, and impact of each difficulty type. This would enable researchers to identify which difficulties result in the most prolonged and intense distress. Nor did we ask about the motivation for taking the psychedelic substance, which some researchers have suggested can predict difficulties [52].

We decided to conduct a fine-grained thematic analysis with a large number of themes, to ensure that the full range and nuance of the experiences was captured—we ended up with 60, which as it happens is almost the same number of themes as a celebrated study of adverse meditative experiences [53], from which we took inspiration. With such a diverse range of themes and nuanced distinctions among them (for example, differentiating between ’fear of dying’ and ’fear of hell’), achieving cross-analyst agreement is a complex task. It took the analyst team three attempts to reach an 80% agreement level. Future studies may consider adopting a more streamlined thematic structure to ensure a more reliable and replicable coding system, which is a vital aspect to take into account. Other teams studying this topic may identify different themes to ours, though we anticipate broad applicability. Many themes emerged that warrant future study, including the high prevalence of anxiety, the nature and phenomenology of de-realization, continued hallucinations and their relationship to flashbacks, the sense of disconnection from others, and the experience of being hurt by others during or directly after the experience, which includes by those leading guided psychedelic sessions.

### Wider and practical implications

Our findings point to a variety of profoundly challenging experiences that can occur following the use of psychedelics, and which can occur following use in both guided and unguided settings. Given that anxiety and fear are some of most common kinds of difficulties, we propose that all legal providers of psychedelic experiences provide a guidance document on methods for self-soothing and overcoming bouts of anxiety following the retreat, clinical trial or ceremony. The variety of possible adverse outcomes presents a challenge for informed consent for psychedelic clinics and retreat centers. Patients and participants should be informed what sorts of harms can occur, the relative risks of these, and advised that the integration process can take months. They could also be informed about some of the practices that help people cope with difficulties, which we explore in a forthcoming paper. We envisage using the information in this study, and accompanying future papers that focus on social support and forms of coping used by those with enduring difficulties, to provide structured guidance and training to psychedelic retreats, therapists and clinical trial centers about the potential for adverse experiences, what the potential risk factors are and what can be do to help individuals who report such extended difficulties.

In the wake of two decades of resurgent psychedelic research, and largely positive media coverage, many people may expect psychedelics to yield only benefits—heightened social connection, reduced anxiety and depression, an enhanced sense of meaning, and a deeper connection to the divine or the universe. However, our survey reveals that psychedelics’ effects can be a double-edged sword for some. These substances can foster greater social connection or, conversely, provoke intense social disconnection. They can alleviate anxiety or exacerbate it. They have the potential to heal post-traumatic stress disorder (PTSD) or induce it. They can boost an individual’s sense of meaning or plunge them into existential confusion. Importantly, these effects are not always transient. Our study is the first to show the range of difficulties people can experience after trips, and how that these difficulties can last months or years. With millions of people turning to psychedelics to healing and personal growth, it is urgent to factor in support for individuals who encounter enduring difficulties. Many of our respondents reported that their difficulties were exacerbated by a lack of information about what was happening to them, which increased their anxiety, feelings of social isolation, and existential confusion. We hope that providing better information about these difficulties is helpful to people in distress and call for more research on adverse psychedelic experiences and what helps people cope with them.
